# Trajectories of lifestyle patterns from 2 to 8 years of age and cardiometabolic risk in children: the GUSTO study

**DOI:** 10.1186/s12966-024-01564-z

**Published:** 2024-01-26

**Authors:** Airu Chia, Jia Ying Toh, Padmapriya Natarajan, Shirong Cai, Yi Ying Ong, Alexandra Descarpentrie, Sandrine Lioret, Jonathan Y Bernard, Falk Müller-Riemenschneider, Keith M Godfrey, Kok Hian Tan, Yap Seng Chong, Johan G Eriksson, Mary F-F Chong

**Affiliations:** 1 Saw Swee Hock School of Public Health, National University of Singapore and National University Health System, Tahir Foundation Building, 12 Science Drive 2, #12 − 01, Singapore, 117549 Singapore; 2 Singapore Institute for Clinical Sciences (SICS), Agency for Science, Technology and Research (A*STAR), Singapore, Singapore; 3 Department of Obstetrics and Gynaecology and Human Potential Translational Research Programme, Yong Loo Lin School of Medicine, National University of Singapore, Singapore, Singapore; 4 Department of Social and Behavioural Sciences, Harvard T.H. Chan School of Public Health, Boston, MA USA; 5 Centre for Research in Epidemiology and Statistics (CRESS), Université Paris Cité and Université Sorbonne Paris Nord, INRAE, Paris, F-75004 France; 6 Digital Health Center, Berlin Institute of Health, Charité-Universitätsmedizin Berlin, Berlin, Germany; 7 MRC Lifecourse Epidemiology Centre and NIHR Southampton Biomedical Research Centre, University of Southampton and University Hospital Southampton NHS Foundation Trust, Southampton, UK; 8 Duke-NUS Medical School, Singapore, Singapore; 9 Department of Maternal Fetal Medicine, KK Women’s and Children’s Hospital, Singapore, Singapore; 10 Department of General Practice and Primary Health Care, University of Helsinki and Helsinki University Hospital, Helsinki, Finland; 11 Folkhälsan Research Centre, Helsinki, Finland

**Keywords:** Longitudinal, Group-based trajectory, Lifestyle patterns, Diet, Physical activity, Screen time, Sleep, Cardiometabolic, Metabolic syndrome, Childhood

## Abstract

**Background:**

Tracking combinations of lifestyle behaviours during childhood (“lifestyle pattern trajectories”) can identify subgroups of children that might benefit from lifestyle interventions aiming to improve health outcomes later in life. However, studies on the critical transition period from early to middle childhood are limited. We aimed to describe lifestyle patterns trajectories in children from 2 to 8 years of age and evaluated their associations with cardiometabolic risk markers at age 8 years in a multi-ethnic Asian cohort.

**Methods:**

Twelve lifestyle behaviours related to child’s diet, physical activity, screen use, and sleep were ascertained using questionnaires at ages 2, 5, and 8 years. Age-specific lifestyle patterns were derived using principal component analysis and trajectories were determined using group-based multi-trajectory modelling. Child cardiometabolic risk markers were assessed at age 8 years, and associations with trajectories examined using multiple regression, adjusted for confounders.

**Results:**

Among 546 children, two lifestyle patterns “healthy” and “unhealthy” were observed at ages 2, 5, and 8 years separately. Three trajectory groups from 2 to 8 years were identified: consistently healthy (11%), consistently unhealthy (18%), and mixed pattern (71%). Children in the consistently unhealthy group (vs. mixed pattern) had increased odds of pre-hypertension (OR = 2.96 [95% CI 1.18–7.41]) and higher levels of diastolic blood pressure (β = 1.91 [0.27–3.55] mmHg), homeostasis model assessment of insulin resistance (β = 0.43 [0.13–0.74]), triglycerides (β = 0.11 [0.00-0.22] mmol/L), and metabolic syndrome score (β = 0.85 [0.20–1.49]), but not with BMI z-score or any anthropometric measurements. The consistently healthy group showed no differences in cardiometabolic outcomes compared to the mixed pattern group.

**Conclusion:**

Three distinct lifestyle pattern trajectories were identified from early to middle childhood. Children in the consistently unhealthy lifestyle group did not have a raised BMI but was associated with several elevated cardiometabolic risk markers. These findings suggest the potential benefits of initiating holistic lifestyle interventions to improve children’s health and well-being from an early age.

**Trial registration:**

Trial registration number: NCT01174875. Name of registry: ClinicalTrials.gov. URL of registry: https://classic.clinicaltrials.gov/ct2/show/NCT01174875. Date of registration: August 4, 2010. Date of enrolment of the first participant to the trial: June 2009.

**Supplementary Information:**

The online version contains supplementary material available at 10.1186/s12966-024-01564-z.

## Background

The rising prevalence of adverse cardiometabolic profiles in children is concerning due to long-term health implications [1]. A meta-analysis of prospective cohort studies suggests that a holistic approach adopting multiple healthy lifestyle behaviours (e.g., better diet quality, increased physical activity, decreased sedentary time, and optimal sleep) acts synergistically to reduce cardiovascular risk, particularly in young adults [2]. However, these studies typically assess lifestyle behaviours at a single time point without quantifying the risk associated with lifestyle changes (or trajectories) over time.

Lifestyle changes can occur at any stage of life, even within the childhood period [3]. Establishment of lifestyle habits often happens before 3 years of age [3]. A sedentary-snacking pattern, characterized by high consumption of energy-dense or processed foods coupled with excessive screen time, is frequently observed and established as early as age 18 months [4–6]. When children enter primary school, around the age of 6–7 years, their lifestyle behaviours may remain stable or undergo changes due to peer influence and other environmental factors [3, 7]. Studying how combinations of lifestyle behaviours track over childhood (also known as lifestyle pattern trajectories) provides valuable insights into subgroups of children that might benefit from lifestyle interventions to improve health, including metabolic outcomes, later in life.

A growing number of studies have adopted the group-based multi-trajectory modelling approach [8, 9]. This method enables the identification of groups of children that follow similar lifestyle trajectories across multiple behaviours of interest. However, existing studies on trajectories of multiple lifestyle behaviours in children have primarily focused on either early childhood (4 to 60 months) [8, 9] or middle childhood to adolescence (7 to 19 years of age) [10–14], with limited research on the critical transition period from early to middle childhood [15, 16]. Furthermore, these studies predominantly focused on movement behaviours, such as physical activity and screen time, but did not study diet and movement behaviours collectively.

While most existing studies aim to characterise lifestyle pattern trajectories and identify their correlates [8–12, 16], fewer relate to risk markers for health outcomes [13–15]. Only one study in the UK observed that children following trajectories of high moderate-to-vigorous intensity physical activity and low sedentary behaviour from 7 to 15 years of age had lower fat mass index in late adolescence [14]. However, no previous studies have assessed associations with cardiometabolic risk markers like insulin sensitivity and paediatric metabolic syndrome score during childhood, which may provide further insights on the early origins of cardiometabolic diseases.

To address these gaps, we aimed to identify distinct lifestyle pattern trajectories from early to middle childhood (ages 2 to 8 years) and examine their associations with cardiometabolic risk markers at age 8 years in a multi-ethnic Asian cohort.

## Methods

### Study population

We studied children from the Growing Up in Singapore Towards healthy Outcomes (GUSTO) prospective mother-offspring cohort study. Briefly, 1450 pregnant women were recruited at 7 to 11 weeks of pregnancy from two public maternity units in Singapore between 2009 and 2010 [17]. Inclusion criteria were women aged 18 years and above, Singapore Citizens or Singapore Permanent Residents, willingness to donate cord, cord blood, and placenta, intending to deliver in the study hospitals and reside in Singapore for the next 5 years, and foetus with both sets of grandparents of the same ethnicity. Women on chemotherapy or with significant health conditions, such as type 1 diabetes and psychosis, were excluded. The study was approved by the National Health Care Group Domain Specific Review Board (D/09/021 and B/2014/00406) and the SingHealth Centralized Institutional Review Board (2018/2767/D and 2009/291/D). Informed written consent was obtained from all participants.

### Lifestyle behaviours

We considered 12 lifestyle behaviours related to the child’s diet and movement behaviours based on existing studies examining lifestyle trajectories in children [8–16]. Movement behaviours include screen time, outdoor play, MVPA, participation in organized physical activity, and sleep duration.

#### Dietary intake

Caregivers (mostly mothers) reported the frequency of food items consumed by their child over the past month using validated food frequency questionnaires at ages 1.5 [18], 5 [19], and 7 years [20]. The data were converted to daily frequencies and categorized into seven food or drink groups, namely fruit, vegetables, processed meat, fast food, sweet snacks, savoury snacks, and sugar sweetened beverages (SSBs) at each time point.

#### Movement behaviours

At age 2 years, caregivers reported the amount of time their child spent on screen use (television, computers, mobile devices, and gaming consoles) and outdoor play on a typical weekday and weekend day over the past month [21, 22]. At ages 5.5 and 8 years, caregivers reported the amount of time their child spent on screen use, outdoor play, and MVPA on the most recent weekday and weekend day using validated preschool-age and school-age physical activity questionnaire [23]. The reported durations for weekdays and weekends were then weighted to calculate the daily average durations. Caregivers also reported the amount of time their child spent on organized physical activity during the week at age 5.5 and 8 years. Outdoor play at age 2 years was used as a surrogate marker for physical activity since there were no MVPA and organized physical activity data at such a young age. This proxy marker is commonly used in the literature [8].

At age 1.5 years, mothers reported day and night sleep durations of their child through the Brief Infant Sleep Questionnaire [24]. At ages 5.5 and 8 years, night sleep duration was reported on a typical weekday and weekend day over the past week through the Child Sleep Habits Questionnaire, weighted to an average day subsequently [25].

#### Identifying patterns of lifestyle behaviours

Principal component analysis (PCA) with varimax rotation was applied to derive lifestyle patterns at ages 2, 5, and 8 years separately. The method is detailed elsewhere [26]. In brief, PCA is a data reduction method that combines correlated standardised variables (here: the 12 lifestyle behaviours) into a smaller set of patterns (here: lifestyle patterns), while preserving as much information as possible. The Kaiser-Meyer-Olkin test statistics for sampling adequacy at Year 2, Year 5, and Year 8 were 0.70, 0.63, and 0.66, respectively. These indicate that the sampling was adequate for PCA. In addition, the Bartlett’s test of sphericity produced a statistically significant chi-square value to justify the application of PCA.

Two consistent lifestyle patterns were identified (See Supplementary Table 1, Additional File 1) and they were subsequently used to derive trajectories. Based on absolute PCA loadings > 0.20, the first pattern was characterized by high intakes of processed meat, fast food, sweet snacks, savoury snacks, SSBs, and screen time. The second pattern was characterized by high intakes of fruit and vegetables, low screen time, and high MVPA, outdoor play, and participation in organized physical activity. The pattern scores for each child was calculated by summing the standardized values of each lifestyle behaviour weighted to its PCA loading. A higher score on the first pattern suggests a less healthy lifestyle, while a higher score on the second pattern suggests a healthier lifestyle. For simplicity, we named them as “unhealthy” and “healthy” patterns.

### Cardiometabolic outcomes at age 8 years

#### Anthropometrics

Trained research staff measured the standing height (seca 213 Stadiometer, seca GmbH & Co., Hamburg, Germany), weight (seca 803 weighing scale), abdominal circumference (measuring tape), and skinfold thicknesses (triceps, biceps, subscapular, and suprailiac skinfolds) from the right side of the body (Holtain skinfold calipers, Holtain Ltd, Pembrokeshire, UK). We calculated the sum of skinfold thicknesses measured at the four sites and derived the sex- and age-specific BMI z-scores using the World Health Organization growth standards [27].

#### Blood pressure

Peripheral systolic and diastolic blood pressure were measured from the right upper arm (Dinamap CARESCAPE V100, GE Healthcare, Milwaukee, WI). An average of two readings was calculated if the difference between readings was less than 10 mmHg; otherwise, a third reading was taken and the average of the three readings used instead. Pre-hypertension for children was defined as ≥ 110/70 mmHg, which has been shown to predict the risk of adult hypertension and subclinical cardiovascular disease [28].

#### Laboratory measures

Fasting blood was drawn to measure plasma glucose (Abbott Architect c8000 analyzer at KK Women’s and Children’s Hospital, and Beckman AU5800 analyzer at National University Hospital), serum insulin (Beckman DXL800 analyzer, Beckman Coulter, Brea, CA), high density lipoprotein cholesterol (Beckman AU5800 analyzer), triglycerides, and gamma-glutamyl-transferase (Beckman AU5800 analyzer). Homeostasis model assessment of insulin resistance (HOMA-IR) was calculated as follows [29]: fasting insulin (mU/L) * fasting glucose (mmol/L) / 22.5.

#### Prediction indices

Paediatric metabolic syndrome score was calculated based on a published approach using sex- and cohort-specific z-scores of abdominal circumference, systolic blood pressure, diastolic blood pressure, HOMA-IR, triglycerides, and high density lipoprotein cholesterol [30].

Fatty liver index was calculated based on a published algorithm using BMI, abdominal circumference, triglycerides, and gamma-glutamyl-transferase [31]. The index has been validated against ultrasonography and has shown good performance for predicting fatty liver.

### Covariates

*A priori* determined covariates were selected as they have been identified as correlates of lifestyle behaviours [8, 9, 12, 15] and/or cardiometabolic outcomes in childhood [32, 33]. A detailed description of the covariates is presented in Supplementary Information, Additional File 1. In brief, covariates were either obtained from clinical records or collected by questionnaires administered during recruitment (7–11 weeks’ gestation), pregnancy (26–28 weeks’ gestation) and at different stages of the follow-up (from ages 3 months to 8 years). They were subsequently categorized into maternal, paternal, pregnancy, offspring, and postnatal characteristics.

### Statistical analysis

We used group-based multi-trajectory modelling to identify lifestyle patterns trajectories across three time points at ages 2, 5, and 8 years [34]. Participants who had missing data for more than one time point were excluded. The optimal number of latent classes and trajectory shapes (i.e. linear, quadratic, cubic) were selected based on Bayesian Information Criteria, adequate sample size in each class, odds of correct classification, model parsimony, and distinctiveness and interpretability of trajectories via visual inspection. The model fit statistics for various trajectory shapes within 2- to 4-class solutions were shown in Supplementary Table 2, Additional File 1.

To understand the profile of children in the various trajectories, covariates (i.e., maternal, paternal, pregnancy, offspring, and postnatal characteristics) were summarised by trajectory groups. Fisher’s exact test and ANOVA were used to compare the differences in characteristics across trajectory groups.

Associations between lifestyle pattern trajectories and cardiometabolic outcomes were performed by linear regression for continuous outcomes and logistic regression for pre-hypertension. We adjusted for factors associated with both lifestyle behaviours and cardiometabolic outcomes in childhood (maternal age, ethnicity, educational attainment, household income, family history of cardiovascular disease, pre-pregnancy BMI, father’s BMI, child sex, birth order, preterm birth,). Blood pressure analyses were further adjusted for child height at the age of 8 years.

The percentage missing on confounders ranged from 0.4 to 9% and they were imputed using chained equations which included all exposures, outcomes, and covariates as predictors. We generated 20 imputed datasets and results of the pooled analyses were presented. We had missing data for cardiometabolic outcomes ranging from 2.9 to 30% owing to differential consent for anthropometrics, blood pressure, and laboratory measurements. Complete case and outcome analyses (participants without missing confounders and outcomes, *n* = 294) were performed as sensitivity analysis.

All statistical analyses were carried out using Stata 17 (StataCorp LP, USA). *P* < 0.05 was considered statistically significant.

## Results

**Fig. 1:**
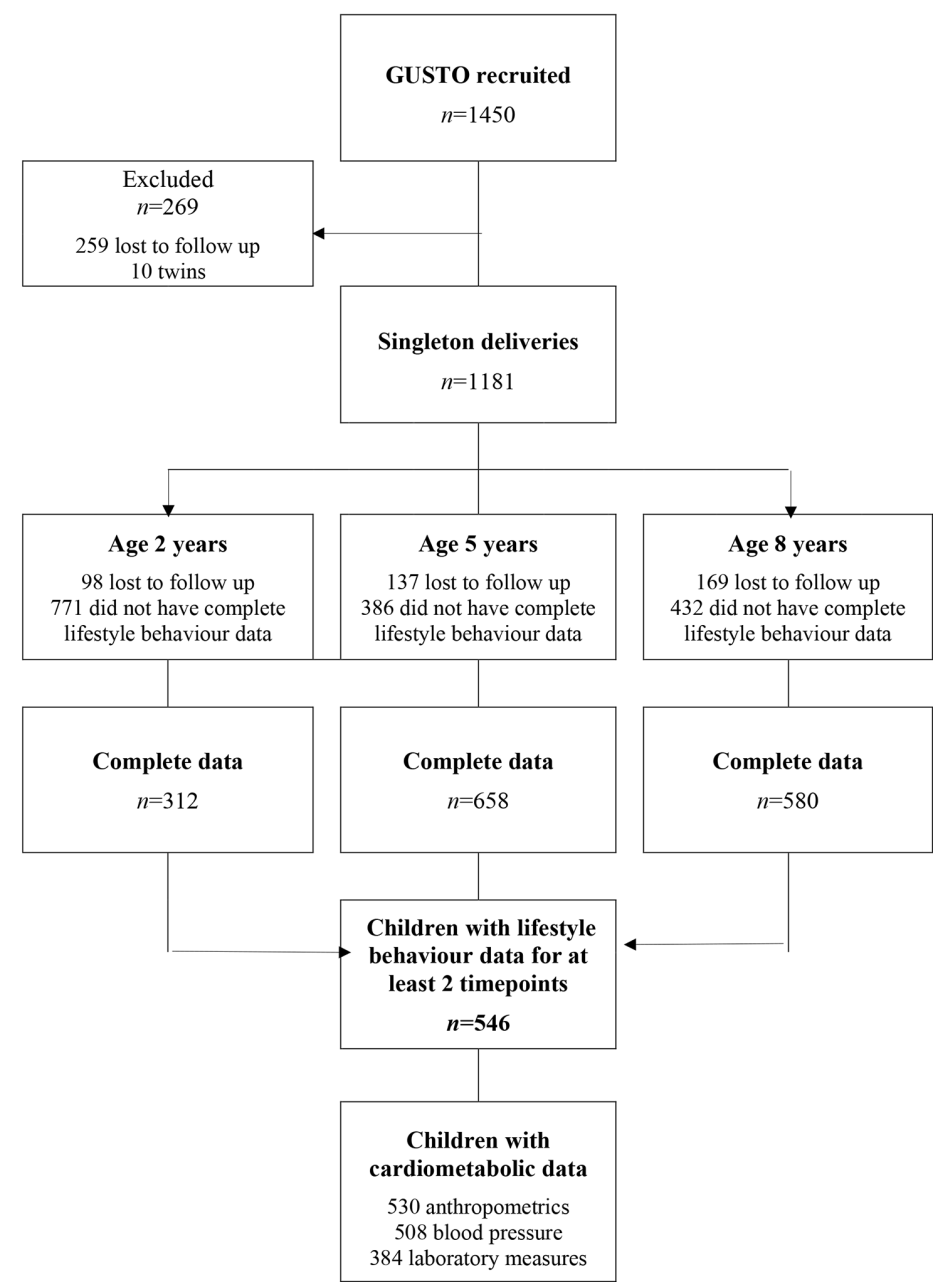
Flowchart of participants

Among 1181 singleton deliveries, 169 children were lost to follow-up and 466 had incomplete lifestyle behaviour data for more than one timepoint, resulting in 546 children for the present analysis (Fig. 1). Children excluded from the analysis did not differ by sex, birth order, or birth size, however, they were more likely of Indian ethnicity, born preterm, to younger and less educated mothers (See Supplementary Table 3, Additional File1).

### Lifestyle pattern trajectories

**Fig. 2:**
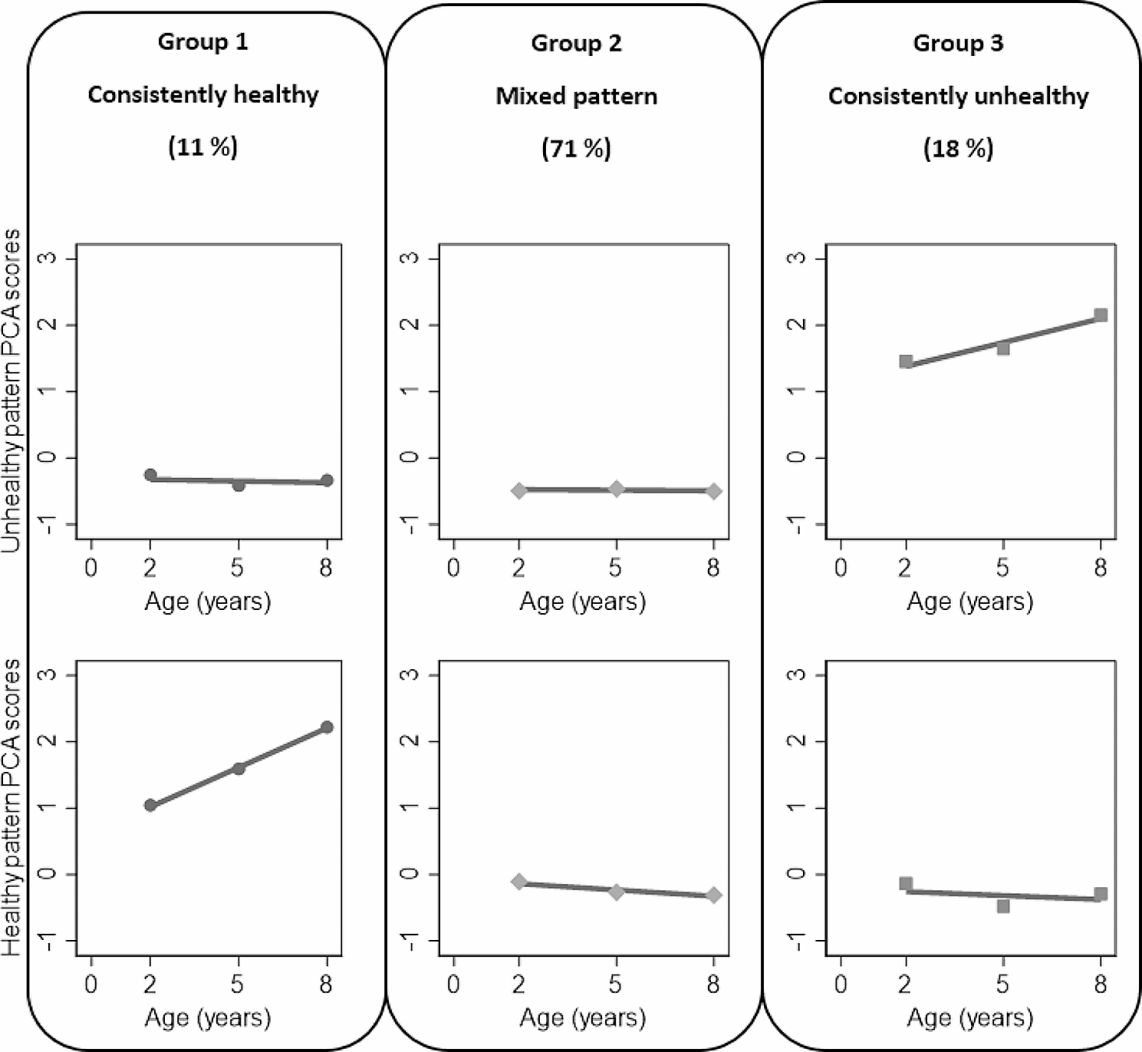
Identified three trajectory groups from two lifestyle patterns. Group 1: Consistently healthy; Group 2: Mixed pattern; Group 3: Consistently unhealthy. Abbreviations: PCA, Principal component analysis. Unhealthy pattern was characterized by high intakes of processed meat, fast food, sweet snacks, savoury snacks, sugar sweetened beverages, and screen time. Healthy pattern was characterized by high intakes of fruit and vegetables, low screen time, and high moderate-to-vigorous physical activity, outdoor play, and participation in organized physical activity. The consistently healthy group maintained a stable-low level for the unhealthy pattern, and a high-increasing trend for the healthy pattern. The consistently unhealthy group displayed a stable-low level for the healthy pattern, and a high-increasing trend for the unhealthy pattern. The mixed pattern revealed a stable-low trajectory for both healthy and unhealthy patterns

We identified three distinct lifestyle pattern trajectories from 2 to 8 years of age: consistently healthy (11%), consistently unhealthy (18%), and mixed pattern (71%) (Fig. 2). The consistently healthy group maintained a stable-low level for the unhealthy pattern and a high-increasing trend for the healthy pattern throughout the 6-year follow-up. Conversely, the consistently unhealthy group displayed a stable-low level for the healthy pattern and a high-increasing trend for the unhealthy pattern.

The mixed trajectory exhibited stable-low level for both patterns. A comparison of individual lifestyle behaviours by trajectory groups revealed that children in this group had lower intakes of fruit and vegetables and were less active compared to the consistently healthy group. However, they consumed less discretionary foods and had lower screen time compared to the consistently unhealthy group (See Supplementary Table 4, Additional File 1).

### Characteristics of study population by trajectory groups

Children in the consistently unhealthy group tended to be of Malay ethnicity, had shorter breastfeeding duration, had parents with lower educational attainment and lower household income, and had mothers who exhibited suboptimal lifestyle during pregnancy (i.e., poor diet, physically inactive, poor sleep quality, higher television viewing time, and higher tobacco exposure). Conversely, children in the consistently healthy group tended to be breastfed longer, had higher proportion of parents with university degrees and higher household income, and born to mothers who had more optimal lifestyle during pregnancy (Table 1).

**Table 1:** Maternal, paternal, pregnancy, offspring, and postnatal characteristics across the trajectory groups (*n* = 546)

	Consistently healthy *n* = 59	Mixed pattern *n* = 390	Consistently unhealthy *n* = 97	p-value
**Maternal characteristics**				
Age at recruitment (years)	31.8 ± 4.8	31.4 ± 4.9	30.1 ± 5.8	0.05
Pre-pregnancy BMI (kg/m^2^)	21.9 ± 3.6	22.6 ± 4.4	23.2 ± 4.4	0.22
Family history of cardiovascular disease	14 (24)	115 (29)	32 (33)	0.47
Ethnicity				**< 0.001**
Chinese	39 (66)	264 (68)	31 (32)	
Malay	1 (1.7)	84 (22)	53 (55)	
Indian	19 (32)	42 (11)	13 (13)	
Educational attainment				**< 0.001**
Secondary and below	7 (12)	98 (25)	36 (37)	
Post-secondary	15 (26)	138 (35)	40 (41)	
University and above	36 (62)	153 (39)	21 (22)	
Household monthly income (S$)				**0.01**
≤ 1999	7 (12)	41 (11)	17 (18)	
2000–5999	23 (40)	199 (54)	58 (62)	
≥ 6000	27 (47)	131 (35)	19 (20)	
**Paternal characteristics**				
Age at recruitment (years)	34.4 ± 5.0	34.7 ± 5.8	34.0 ± 6.6	0.62
BMI (kg/m^2^)	24.7 ± 3.5	25.7 ± 4.5	26.4 ± 4.7	0.08
Educational attainment				**< 0.001**
Secondary and below	4 (8.0)	69 (22)	24 (29)	
Post-secondary	14 (28)	104 (32)	39 (47)	
University and above	32 (64)	148 (46)	20 (24)	
**Pregnancy characteristics**				
Diet quality (HEI-SGP)	59.8 ± 13.5	53.5 ± 13.6	48.4 ± 13.2	**< 0.001**
MVPA ≥ 150 min/week	11 (19)	27 (7.1)	8 (8.4)	**0.02**
Television viewing time < 2 h/day	38 (64)	182 (47)	33 (35)	**0.001**
Good sleep quality (PSQI < 5)	29 (74)	144 (59)	16 (31)	**0.001**
Tobacco exposure (plasma cotinine > LOD)	3 (5.7)	52 (15)	28 (31)	**0.001**
Gestational diabetes mellitus	14 (26)	71 (19)	11 (12)	0.09
Depressive symptoms (EPDS ≥ 15)	2 (3.4)	22 (5.8)	10 (11)	0.14
Anxiety symptoms (STAI ≥ 42)	6 (10)	83 (22)	27 (30)	**0.02**
Rate of gestational weight gain				0.10
Inadequate	5 (9.4)	56 (16)	9 (11)	
Normal	29 (55)	124 (36)	30 (37)	
Excessive	19 (36)	166 (48)	42 (52)	
**Offspring characteristics**				
Boy	33 (56)	199 (51)	53 (55)	0.69
Firstborn	25 (42)	187 (48)	33 (34)	**0.04**
Preterm birth	4 (6.8)	20 (5.1)	7 (7.2)	0.60
Size for gestational age				0.68
Appropriate for gestational age	44 (75)	275 (71)	69 (71)	
Small for gestational age	5 (8.5)	55 (14)	10 (10)	
Large for gestational age	10 (17)	60 (15)	18 (19)	
**Postnatal nutrition**				
Duration of any breastfeeding				**0.002**
< 1 month	4 (7.1)	68 (18)	27 (29)	
1–5 months	20 (36)	148 (39)	40 (43)	
≥ 6 months	32 (57)	163 (43)	27 (29)	
Early complementary feeding (≤ 4 months)	7 (14)	58 (17)	21 (26)	0.16

Abbreviations: EPDS, Edinburgh Postnatal Depression Scale; HET-SGP, Healthy Eating Index for pregnant women in Singapore; LOD, Limit of detection; MVPA, moderate-to-vigorous intensity physical activity; PSQI, Pittsburgh Sleep Quality Index; STAI, State-Trait Anxiety Inventory

^1^Values are means ± SDs or n(%). P-values were obtained using Fisher’s exact test for categorical variables and ANOVA for continuous variables

### Association of childhood lifestyle pattern trajectories and cardiometabolic outcomes

After adjusting for confounders, children in the consistently unhealthy group (vs. mixed pattern) had increased odds of pre-hypertension (OR = 2.96 [95% CI 1.18–7.41]) and higher levels of diastolic blood pressure (β = 1.91 [0.27–3.55] mmHg), fasting insulin (β = 13.7 [4.24–23.2] pmol/L), HOMA-IR (β = 0.43 [0.13–0.74]), triglycerides (β = 0.11 [0.00-0.22] mmol/L), and metabolic syndrome score (β = 0.85 [0.20–1.49]), but not with any anthropometric measurements (Table 2). The consistently healthy group showed no differences in cardiometabolic outcomes compared to the mixed pattern group (Table 2).

**Table 2:** Associations between lifestyle pattern trajectories and child cardiometabolic risk markers at the age of 8 years (*n* = 546)^1^

	Unadjusted				Adjusted^2^				
	Consistently healthy		Consistently unhealthy		Consistently healthy		Consistently unhealthy		
	B (95% CI)	P	B (95% CI)	P	B (95% CI)	P	B (95% CI)	P	
**Anthropometrics**									
BMI z-score (SDS)	0.10 (-0.30, 0.50)	0.62	0.02 (-0.31, 0.35)	0.91	0.27 (-0.13, 0.68)	0.19	-0.03 (-0.37, 0.31)	0.87	
Abdominal circumference (cm)	0.70 (-1.62, 3.02)	0.55	0.26 (-1.68, 2.20)	0.79	0.60 (-1.76, 2.95)	0.62	0.30 (-1.68, 2.28)	0.77	
Sum of skinfolds (mm)	-0.06 (-5.08, 4.97)	0.98	0.34 (-3.86, 4.54)	0.88	0.57 (-4.49, 5.64)	0.83	0.27 (-4.01, 4.55)	0.90	
**Blood pressure**									
Systolic (mmHg)	-0.14 (-2.75, 2.46)	0.92	1.76 (-0.36, 3.88)	0.10	0.06 (-2.51, 2.64)	0.96	2.10 (-0.03, 4.24)	0.05	
Diastolic (mmHg)	0.65 (-1.24, 2.54)	0.50	1.38 (-0.15, 2.92)	0.08	0.39 (-1.59, 2.37)	0.70	1.91 (0.27, 3.55)	**0.02**	
Pre-hypertension^3^	0.89 (0.26, 3.09)	0.86	2.16 (1.01, 4.63)	**0.05**	1.01 (0.27, 3.85)	0.99	2.96 (1.18, 7.41)	**0.02**	
**Laboratory measures**									
Fasting glucose (mmol/L)	0.02 (-0.09, 0.13)	0.68	-0.02 (-0.11, 0.07)	0.69	-0.02 (-0.13, 0.09)	0.78	0.03 (-0.06, 0.12)	0.54	
Fasting insulin (pmol/L)	0.94 (-9.90, 11.8)	0.87	12.8 (3.85, 21.7)	**0.01**	-2.74 (-14.3, 8.87)	0.69	13.7 (4.24, 23.2)	**0.01**	
HOMA-IR	0.02 (-0.32, 0.37)	0.66	0.39 (0.11, 0.67)	**0.01**	-0.10 (-0.47, 0.27)	0.60	0.43 (0.13, 0.74)	**0.01**	
Triglyceride (mmol/L)	-0.09 (-0.21, 0.04)	0.48	0.12 (0.02, 0.23)	**0.02**	-0.06 (-0.19, 0.08)	0.40	0.11 (0.00, 0.22)	**0.05**	
HDL cholesterol (mmol/L)	0.08 (-0.01, 0.17)	0.09	-0.06 (-0.13, 0.01)	0.11	0.08 (-0.02, 0.17)	0.10	-0.05 (-0.13, 0.03)	0.22	
**Prediction indices**									
Metabolic syndrome score	0.00 (-0.76, 0.77)	0.99	0.84 (0.22, 1.46)	**0.01**	-0.14 (-0.93, 0.65)	0.73	0.85 (0.20, 1.49)	**0.01**	
Fatty liver index	-0.41 (-2.13, 1.31)	0.64	0.71 (-0.70, 2.12)	0.32	0.02 (-1.88, 1.72)	0.93	0.36 (-1.09, 1.82)	0.62	

Abbreviations: HDL, high-density lipoprotein; HOMA-IR, homeostasis model assessment of insulin resistance; SDS, standard deviation score; z-BMI, z-score of body mass index

^1^Multiple linear regression coefficient estimates (95%CI) and p-values are presented, referenced to the mixed trajectory

^2^Models were adjusted for maternal age, ethnicity, educational attainment, household income, family history of cardiovascular disease, pre-pregnancy BMI, father’s BMI, child sex, birth order, and preterm birth. BMI z-score was not additionally adjusted for sex. Blood pressure analyses were further adjusted for child height at the age of 8 years

^3^Binary logistic regression odds ratio (95%CI) and p-values are presented, referenced to the mixed trajectory

Similar direction of associations were found between the consistently healthy and consistently unhealthy groups (Supplementary Table 5) and in complete-case and -outcome analyses (Supplementary Table 6).

## Discussion

To our knowledge, this study is the first to identify lifestyle pattern trajectories from early to middle childhood and cardiometabolic risk factors in children. We found three distinct lifestyle pattern trajectories. Only 11% of the children were categorized in the consistently healthy group, whereas nearly one-fifth belonged to the consistently unhealthy group. The majority exhibited mixed healthy and unhealthy lifestyle pattern trajectories. Children in the consistently unhealthy group had increased odds of pre-hypertension and higher levels of diastolic blood pressure, fasting insulin, HOMA-IR, triglycerides, and metabolic syndrome score at 8 years of age.

The study identified distinct lifestyle pattern trajectories with variability in multiple lifestyle behaviours. Majority of children (71%) exhibited a mixed pattern trajectory, characterised by low physical activity, moderate screen time, and low intakes of fruit, vegetables, and discretionary foods. Consistent with longitudinal data of Spanish youth [11], 75% of the children had low MVPA and slight increase in sedentary behaviour over time. Only one study in Australia examined diet and movement behaviours collectively and they reported that 53% of children belonged to the trajectory group with healthy lifestyle patterns during a follow-up period from 18 to 60 months [9]. This reported percentage is notably higher than our findings (11%). However, it is important to note that the study in Australia primarily focused on early childhood and it is unclear whether the healthy pattern persist into middle childhood when children enter primary school. Further investigations are warranted, particularly in studies with extended follow-up periods, to gain deeper insights into the factors influencing the persistence of healthy lifestyle patterns in children over time.

We noted relatively lower PCA loadings for screen time and sleep variables during the identification of lifestyle patterns, suggesting that screen time and sleep make a smaller contribution to the overall lifestyle pattern. Notably, sleep duration in children has been shown to track poorly [35]. A study involving children aged 3 to 7 years revealed that sleep patterns in children are not particularly stable, showing considerable variation both within a week and across the years.

Children in the consistently unhealthy group exhibited a cluster of cardiometabolic risk markers at 8 years of age, despite having similar BMI and anthropometric measurements as children in the mixed trajectory group, which aligns with the Asian metabolic phenotype [36]. Previous studies have not assessed associations between trajectories of multiple lifestyle behaviours and cardiometabolic risk markers in childhood. Only one study in the UK reported that children with high MVPA and low sedentary time throughout 7 to 15 years of age had lower fat mass index in late adolescence, but no direct relationship with BMI was observed [14]. Studies assessing lifestyle behaviours at a single time point also showed inconsistent BMI results [37]. Younger children are hypothesized to have better energy compensation abilities than older children and adults [38], suggesting that children with unhealthy lifestyle may not always lead to weight gain. Therefore, our study underscores the importance of measuring various cardiometabolic risk markers, alongside BMI, to detect early cardiometabolic risk in children.

It is also noteworthy that certain cardiometabolic risk markers such as fasting glucose and liver fat were not elevated in the consistently unhealthy group; this may be because differences only emerge gradually over time and may be more pronounced after puberty [39]. Nonetheless, our study provides novel insights on the early development of metabolic abnormalities as we found concomitant increases in blood pressure, insulin resistance, triglycerides, and metabolic syndrome score among 8-year-old children. As cardiometabolic risk factor clustering is predictive of future cardiovascular disease [40], our results highlight the clinical significance of promoting healthy behaviours from a young age, especially in children with consistently unhealthy lifestyle behaviours, to prevent adverse metabolic outcomes later in life.

Consistent with previous studies [8, 9, 26], children in the consistently unhealthy group tended to be of Malay ethnicity, had shorter breastfeeding duration, had parents with lower educational attainment and lower household income, and had mothers who exhibited suboptimal lifestyle during pregnancy. The prenatal period has been recognised as a time when women may be more receptive to changing their health behaviours [41]. Targeting positive behaviour change during pregnancy, which often continues after childbirth and integrated into family routines, may be effective in promoting healthier lifestyles for children. Alongside individual-level interventions, it is crucial to incorporate structural measures, such as social and community support, to empower families, particularly those experiencing social disadvantage, to adopt healthy behavioural trajectories.

Strengths of our study include repeated measures of multiple lifestyle behaviours across early and middle childhood, the use of group-based multi-trajectory modelling to summarize complex longitudinal data, and the evaluation of a comprehensive panel of cardiometabolic risk markers at the age of 8 years to capture any early subclinical changes in cardiometabolic profile.

Some study limitations are worth noting. First, data on children’s lifestyle behaviours were caregiver-reported. Nevertheless, measurement errors are more likely non-differential since cardiometabolic outcomes were collected prospectively. Moreover, the tools for assessing these child behaviours have shown good validity and used in past research [18–25]. Second, the small sample size of the consistently healthy group (*n* = 59) may limit the power to detect associations with cardiometabolic risk markers. Third, a significant proportion of children in the mixed pattern trajectory (71%) could also be due to “regression to the mean” [42] where children tended to gravitate towards the mean if behaviours were measured repeatedly over time. Fourth, the evaluation of multiple cardiometabolic outcomes raises the possibility of false-positive results, but the consistency of associations across related outcomes reduces this concern. Fifth, children excluded from the analysis tended to be of Indian ethnicity, born preterm, to younger and less educated mothers. These characteristics may limit generalisability of our study. Sixth, although we adjusted for many confounders, our findings could still be influenced by residual and unmeasured covariates (e.g., environment and social factors). Last, causality cannot be claimed as in any single observational study, but the longitudinal design allowed us to establish temporality between the exposures and the outcomes.

## Conclusions

In conclusion, we identified three distinct lifestyle pattern trajectories from early to middle childhood, with nearly one-fifth of the children belonging to a consistently unhealthy group. These children did not have a raised BMI but exhibited a cluster of cardiometabolic risk markers, characterized by increased odds of pre-hypertension and elevated levels of diastolic blood pressure, markers of insulin resistance, triglycerides, and metabolic syndrome score. Our findings suggest the potential benefits of initiating holistic lifestyle interventions, involving parents and families, to improve children’s health and well-being from an early age.

### Electronic supplementary material

Below is the link to the electronic supplementary material.

Additional File 1

## Data Availability

Data described in the manuscript and analytic code can be made available upon request after approval from our study executives.

## Declarations

FMR and JYB are members of the Editorial Board of International Journal of Behavioral Nutrition and Physical Activity. They were not involved in the journal’s peer review process of, or decisions related to, this manuscript.

## Abbreviations

GUSTO: Growing Up in Singapore Towards healthy Outcomes
HOMA-IR: Homeostasis model assessment of insulin resistance
MVPA: Moderate-to-vigorous physical activity
PCA: Principal component analysis
SSBs: Sugar sweetened beverages
