# Supplementary material for: Trajectories of lifestyle patterns from 2 to 8 years of age and cardiometabolic risk in children: the GUSTO study
Source: Int J Behav Nutr Phys Act. 2024 Jan 26;21:9. doi: 10.1186/s12966-024-01564-z (PMC10811908; doi:10.1186/s12966-024-01564-z)
Supplement: Supplementary file 1 — Additional File 1 [file 12966_2024_1564_MOESM1_ESM.docx]

# Supplementary Table 1: Principal component analysis loadings of lifestyle patterns derived at age 2, 5, and 8 years^1^

|  |  | Age 2 years  (n=312) | |  | Age 5 years  (n=658) | |  | Age 8 years  (n=580) | |
| --- | --- | --- | --- | --- | --- | --- | --- | --- | --- |
|  |  | Unhealthy | Healthy |  | Unhealthy | Healthy |  | Unhealthy | Healthy |
| Vegetables |  |  | 0.61 |  |  | 0.46 |  |  | 0.38 |
| Fruit |  |  | 0.65 |  |  | 0.48 |  |  | 0.46 |
| Processed meat |  | 0.49 |  |  | 0.36 |  |  | 0.45 |  |
| Fast food |  | 0.43 | -0.20 |  | 0.47 |  |  | 0.44 |  |
| Sweet snacks |  | 0.42 |  |  | 0.50 |  |  | 0.46 |  |
| Savoury snacks |  | 0.39 |  |  | 0.42 |  |  | 0.46 |  |
| Sugar sweetened beverages |  | 0.36 |  |  | 0.34 |  |  | 0.31 |  |
| Outdoor play |  |  | 0.30 |  |  | 0.41 |  |  | 0.42 |
| Moderate-to-vigorous physical activity |  | No data | No data |  |  | 0.36 |  |  | 0.49 |
| Organized physical activity |  | No data | No data |  |  | 0.39 |  |  | 0.37 |
| Screen time |  | 0.26 |  |  | 0.26 |  |  | 0.24 | -0.20 |
| Sleep duration |  |  |  |  |  | 0.20 |  |  |  |
|  |  |  | |  |  | |  |  | |

^1^PCA loadings represent correlation coefficients between the behavioural variables and the derived pattern. Absolute values <0.20 were not listed for simplicity.

Unhealthy pattern was characterized by high intakes of processed meat, fast food, sweet snacks, savoury snacks, sugar sweetened beverages, and screen time. Healthy pattern was characterized by high intakes of fruit and vegetables, low screen time, and high moderate-to-vigorous physical activity, outdoor play, and participation in organized physical activity. KMO, Kaiser-Meyer-Olkin .

# Supplementary Table 2: Model fit statistics of the group-based multi-trajectory modelling for 2- to 4-class solutions

| **Number of classes** | **Trajectory Shapes^1^** | **Bayesian Information Criterion** (least negative) | **Relative entropy** (closer to 1) | **Estimated group membership** | **Actual group membership** (> 5%) | **Average posterior probability assignment** (above 0.70) | **Odds of correct classification** (above 5) |
| --- | --- | --- | --- | --- | --- | --- | --- |
| 2 | (3 3) (3 3) | -4333 | 0.80 | 80.9 | 81.7 | 0.96 | 5.7 |
|  |  |  |  | 19.1 | 18.3 | 0.86 | 26.9 |
| 3 | (3 3 3) (3 3 3) | -4281 | 0.79 | 71.1 | 73.0 | 0.92 | 4.5 |
|  |  |  |  | 16.9 | 15.9 | 0.87 | 35.9 |
|  |  |  |  | 12.0 | 11.0 | 0.83 | 40.7 |
| 3 | (2 2 2) (2 2 2) | -4262 | 0.79 | 71.1 | 73.1 | 0.92 | 5.0 |
|  |  |  |  | 16.9 | 15.9 | 0.87 | 33.4 |
|  |  |  |  | 12.0 | 11.0 | 0.83 | 36.9 |
| 3 | (1 1 1) (1 1 1) | -4247 | 0.78 | 11.8 | 10.8 | 0.83 | 36.8 |
|  |  |  |  | 70.0 | 71.4 | 0.93 | 5.3 |
|  |  |  |  | 18.2 | 17.8 | 0.86 | 27.2 |
| 3 | (0 0 0) (0 0 0) | -4238 | 0.76 | 68.7 | 70.3 | 0.92 | 4.92 |
|  |  |  |  | 14.5 | 13.6 | 0.83 | 29.1 |
|  |  |  |  | 16.7 | 16.1 | 0.84 | 26.4 |
| 4 | (3 3 3 3) (3 3 3 3) | -4257 | 0.79 | 22.5 | 22.3 | 0.83 | 17.3 |
|  |  |  |  | 62.5 | 63.2 | 0.91 | 6.1 |
|  |  |  |  | 2.9 | 2.9 | 0.90 | 317.8 |
|  |  |  |  | 12.1 | 11.5 | 0.83 | 35.3 |

^1^0= Intercept, 1=linear, 2=quadratic, 3=cubic

# Supplementary Table 3. Characteristics of included and excluded participants^1^

| Characteristics | Excluded  n=635 | Included  n=546 | P-value |
| --- | --- | --- | --- |
| Maternal age at delivery, y | 30.1 ± 5.1 | 31.2 ± 5.1 | **<0.001** |
| Ethnicity |  |  | **<0.001** |
| Chinese | 328 (52) | 334 (62) |  |
| Malay | 162 (26) | 138 (25) |  |
| Indian | 143 (23) | 74 (14) |  |
| Maternal educational attainment |  |  | **0.001** |
| Secondary and below | 215 (35) | 141 (26) |  |
| Post-secondary | 220 (35) | 193 (35) |  |
| University and above | 186 (30) | 210 (39) |  |
| Boy | 335 (53) | 285 (52) | 0.78 |
| Firstborn | 291 (46) | 245 (45) | 0.72 |
| Preterm birth | 59 (9.3) | 31 (5.7) | **0.02** |
| Size for gestational age |  |  | 0.17 |
| Small-for-gestational age | 97 (16) | 70 (13) |  |
| Appropriate-for-gestational age | 447 (72) | 388 (71) |  |
| Large-for-gestational age | 81 (13) | 88 (16) |  |
| ^1^Values are means ± SD or *n* (%). Chi-square and Independent sample t tests were used to compare frequencies and means, respectively. | | | |

# Supplementary Table 4. Characteristics of lifestyle behaviours across age groups and lifestyle pattern trajectories^1^

|  |  | **Age 2 years** | | |  | **Age 5 years** | | |  | **Age 8 years** | | |
| --- | --- | --- | --- | --- | --- | --- | --- | --- | --- | --- | --- | --- |
|  |  | G1  n=26 | G2  n=188 | G3  n=61 |  | G1  n=57 | G2  n=361 | G3  n=89 |  | G1  n=53 | G2  n=360 | G3  n=85 |
| Vegetables, frequency/day |  | 3.5 (2.4) | 1.7 (1.9) | 1.5 (1.6) |  | 2.4 (1.9) | 1.3 (1.5) | 0.6 (1.3) |  | 1.2 (1.1) | 0.5 (0.7) | 0.5 (0.6) |
| Fruit, frequency/day |  | 1.9 (1.6) | 1.0 (1.2) | 1.1 (1.3) |  | 1.6 (1.1) | 1.0 (0.8) | 0.8 (0.9) |  | 1.7 (1.6) | 0.9 (0.9) | 1.1 (0.9) |
| Processed meat, frequency/day |  | 0.0 (0.2) | 0.0 (0.2) | 0.3 (0.4) |  | 0.2 (0.4) | 0.3 (0.3) | 0.6 (0.6) |  | 0.3 (0.4) | 0.3 (0.4) | 0.9 (0.8) |
| Fast food, frequency/day |  | 0.1 (0.1) | 0.0 (0.1) | 0.2 (0.3) |  | 0.1 (0.1) | 0.1 (0.1) | 0.3 (0.3) |  | 0.2 (0.2) | 0.2 (0.1) | 0.5 (0.4) |
| Sweet snacks, frequency/day |  | 0.9 (1.3) | 0.7 (0.9) | 1.3 (1.9) |  | 1.1 (1.0) | 1.0 (0.9) | 1.8 (1.7) |  | 1.0 (1.2) | 0.9 (0.8) | 2.2 (1.7) |
| Savoury snacks, frequency/day |  | 0.0 (0.1) | 0.0 (0.1) | 0.1 (0.2) |  | 0.0 (0.2) | 0.1 (0.1) | 0.1 (0.4) |  | 0.1 (0.2) | 0.1 (0.1) | 0.4 (0.4) |
| Sugar sweetened beverages, frequency/day |  | 0.6 (0.8) | 0.4 (0.9) | 1.2 (1.6) |  | 1.1 (1.0) | 0.7 (0.9) | 1.4 (1.4) |  | 1.1 (1.2) | 0.8 (0.7) | 1.4 (1.0) |
| Outdoor play, h/day |  | 1.3 (1.6) | 0.6 (0.8) | 0.6 (1.4) |  | 1.2 (1.6) | 0.7 (1.0) | 0.6 (1.1) |  | 1.9 (2.0) | 1.0 (1.4) | 1.0 (1.6) |
| Moderate-to-vigorous physical activity, h/day |  | No data | No data | No data |  | 2.0 (3.7) | 0.8 (1.2) | 1.2 (1.6) |  | 4.5 (3.7) | 1.2 (2.0) | 1.5 (2.2) |
| Organized physical activity, h/week |  | No data | No data | No data |  | 1.5 (3.0) | 0.0 (1.5) | 0.0 (1.0) |  | 2.5 (5.0) | 0.9 (1.5) | 0.0 (1.0) |
| Screen time, h/day |  | 2.5 (2.4) | 1.6 (2.3) | 2.7 (3.2) |  | 0.8 (1.1) | 1.3 (1.7) | 2.4 (2.3) |  | 1.4 (1.4) | 2.2 (2.1) | 3.5 (3.4) |
| Sleep duration, h/day |  | 12 (1.5) | 12 (1.9) | 11 (2.3) |  | 9.9 (1.0) | 9.5 (1.0) | 9.4 (1.3) |  | 9.3 (1.0) | 9.1 (0.8) | 8.9 (1.1) |

Abbreviations: G1, Group 1 Consistently healthy; G2, Mixed lifestyle; G3, Group 3 Consistently unhealthy.

^1^Values are median (IQR). Kruskal-Wallis tests were used to compare the differences across trajectory groups; all p<0.05.

# Supplementary Table 5: Associations between lifestyle pattern trajectories and child cardiometabolic risk markers at the age of 8 years, referenced to the consistently healthy trajectory (n=546)^1^

|  |  | **Unadjusted** | | | | |  | **Adjusted^2^** | | | | |
| --- | --- | --- | --- | --- | --- | --- | --- | --- | --- | --- | --- | --- |
|  |  | Mixed | |  | Consistently unhealthy | |  | Mixed | |  | Consistently unhealthy | |
|  |  | B (95% CI) | P |  | B (95% CI) | P |  | B (95% CI) | P |  | B (95% CI) | P |
| **Anthropometrics** |  |  |  |  |  |  |  |  |  |  |  |  |
| BMI z-score (SDS) |  | -0.10 (-0.50, 0.30) | 0.62 |  | -0.08 (-0.56, 0.40) | 0.74 |  | -0.27 (-0.68, 0.13) | 0.19 |  | -0.30 (-0.80, 0.20) | 0.24 |
| Abdominal circumference (cm) |  | -0.70 (-3.02, 1.62) | 0.55 |  | -0.44 (-3.21, 2.33) | 0.76 |  | -0.60 (-2.95, 1.76) | 0.62 |  | -0.30 (-3.18, 2.58) | 0.84 |
| Sum of skinfolds (mm) |  | 0.06 (-4.97, 5.08) | 0.98 |  | 0.40 (-5.62, 6.41) | 0.90 |  | -0.57 (-5.64, 4.49) | 0.83 |  | -0.29 (-6.49, 5.92) | 0.93 |
| **Blood pressure** |  |  |  |  |  |  |  |  |  |  |  |  |
| Systolic (mmHg) |  | 0.14 (-2.46, 2.75) | 0.92 |  | 1.90 (-1.18, 4.98) | 0.23 |  | -0.06 (-2.64, 2.51) | 0.96 |  | 2.04 (-1.09, 5.17) | 0.20 |
| Diastolic (mmHg) |  | -0.65 (-2.54, 1.24) | 0.50 |  | 0.73 (-1.50, 2.97) | 0.52 |  | -0.39 (-2.37, 1.59) | 0.70 |  | 1.52 (-0.89, 3.93) | 0.22 |
| Pre-hypertension^3^ |  | -0.89 (-3.09, -0.26) | 0.86 |  | 0.88 (-0.44, 2.20) | 0.19 |  | -1.01 (-3.85, -0.27) | 0.99 |  | 1.07 (-0.40, 2.54) | 0.15 |
| **Laboratory measures** |  |  |  |  |  |  |  |  |  |  |  |  |
| Fasting glucose (mmol/L) |  | -0.02 (-0.13, 0.09) | 0.68 |  | -0.04 (-0.17, 0.09) | 0.53 |  | 0.02 (-0.09, 0.13) | 0.78 |  | 0.05 (-0.09, 0.18) | 0.52 |
| Fasting insulin (pmol/L) |  | -0.94 (-11.8, 9.90) | 0.87 |  | 11.8 (-0.90, 24.6) | **0.07** |  | 2.74 (-8.87, 14.3) | 0.69 |  | 16.5 (2.50, 30.5) | **0.02** |
| HOMA-IR |  | -0.02 (-0.37, 0.32) | 0.66 |  | 0.37 (-0.04, 0.77) | 0.08 |  | 0.10 (-0.27, 0.47) | 0.60 |  | 0.54 (0.09, 0.98) | **0.02** |
| Triglyceride (mmol/L) |  | 0.09 (-0.04, 0.21) | 0.48 |  | 0.21 (0.06, 0.36) | **0.01** |  | 0.06 (-0.08, 0.19) | 0.40 |  | 0.17 (0.01, 0.33) | **0.04** |
| HDL cholesterol (mmol/L) |  | -0.08 (-0.17, 0.01) | 0.09 |  | -0.14 (-0.24, -0.03) | 0.10 |  | -0.08 (-0.17, 0.02) | 0.10 |  | -0.13 (-0.24, -0.01) | **0.03** |
| **Prediction indices** |  |  |  |  |  |  |  |  |  |  |  |  |
| Metabolic syndrome score |  | 0.00 (-0.77, 0.76) | 0.99 |  | 0.84 (-0.05, 1.73) | 0.07 |  | 0.14 (-0.65, 0.93) | 0.73 |  | 0.99 (0.04, 1.94) | **0.04** |
| Fatty liver index |  | 0.41 (-1.31, 2.13) | 0.64 |  | 1.12 (-0.90, 3.14) | 0.28 |  | -0.02 (-1.72, 1.88) | 0.93 |  | 0.46 (-1.69, 2.61) | 0.68 |

Abbreviations: HDL, high-density lipoprotein; HOMA-IR, homeostasis model assessment of insulin resistance; SDS, standard deviation score; z-BMI, z-score of body mass index.

^1^Multiple linear regression coefficient estimates (95%CI) and p-values are presented, referenced to the consistently healthy trajectory.

^2^Models were adjusted for maternal age, ethnicity, educational attainment, household income, family history of cardiovascular disease, pre-pregnancy BMI, father’s BMI, child sex, birth order, and preterm birth. BMI z-score was not additionally adjusted for sex. Blood pressure analyses were further adjusted for child height at the age of 8 years.

^3^Binary logistic regression odds ratio (95%CI) and p-values are presented, referenced to the consistently healthy trajectory.

# Supplementary Table 6. Associations between lifestyle patterns trajectories and child cardiometabolic risk markers at the age of 8 years (n=294) ^1^

|  |  | **Complete case and outcome analysis** | | | | |
| --- | --- | --- | --- | --- | --- | --- |
|  |  | Consistently healthy | |  | Consistently unhealthy | |
|  |  | B (95% CI) | P |  | B (95% CI) | P |
| **Anthropometrics** |  |  |  |  |  |  |
| BMI z-score (SDS) |  | 0.24 (-0.27, 0.74) | 0.35 |  | 0.05 (-0.38, 0.48) | 0.83 |
| Abdominal circumference (cm) |  | -0.11 (-3.04, 2.81) | 0.94 |  | 1.15 (-1.34, 3.64) | 0.36 |
| Sum of skinfolds (mm) |  | -0.92 (-7.17, 5.34) | 0.77 |  | 0.52 (-4.82, 5.86) | 0.85 |
| **Blood pressure** |  |  |  |  |  |  |
| Systolic (mmHg) |  | 0.69 (-2.40, 3.77) | 0.66 |  | 1.69 (-0.94, 4.31) | 0.21 |
| Diastolic (mmHg) |  | 0.36 (-2.05, 2.78) | 0.78 |  | 1.62 (-0.44, 3.68) | 0.12 |
| Pre-hypertension^2^ |  | 1.25 (0.27, 5.72) | 0.78 |  | 4.69 (1.30, 16.9) | 0.02 |
| **Laboratory measures** |  |  |  |  |  |  |
| Fasting glucose (mmol/L) |  | -0.04 (-0.17, 0.09) | 0.56 |  | 0.00 (-0.11, 0.10) | 0.93 |
| Fasting insulin (pmol/L) |  | -4.89 (-14.9, 5.10) | 0.34 |  | 4.43 (-4.06, 12.9) | 0.31 |
| HOMA-IR (units) |  | -0.17 (-0.48, 0.15) | 0.29 |  | 0.14 (-0.13, 0.40) | 0.32 |
| Triglyceride (mmol/L) |  | -0.05 (-0.21, 0.10) | 0.48 |  | 0.12 (-0.01, 0.25) | 0.07 |
| HDL cholesterol (mmol/L) |  | 0.09 (-0.01, 0.19) | 0.08 |  | -0.02 (-0.11, 0.06) | 0.58 |
| **Prediction indices** |  |  |  |  |  |  |
| Metabolic syndrome score |  | -0.31 (-1.14, 0.53) | 0.47 |  | 0.65 (-0.06, 1.36) | 0.07 |
| Fatty liver index |  | -0.17 (-1.51, 1.18) | 0.81 |  | 0.77 (-0.38, 1.91) | 0.19 |

Abbreviations: HDL, high-density lipoprotein; HOMA-IR, homeostasis model assessment of insulin resistance; SDS, standard deviation score; z-BMI, z-score of body mass index.

^1^Multiple linear regression coefficient estimates (95%CI) and p-values are presented, referenced to the mixed trajectory. Models were adjusted for maternal age, ethnicity, educational attainment, household income, family history of cardiovascular disease, pre-pregnancy BMI, father’s BMI, child sex, birth order, and preterm birth. BMI z-score was not additionally adjusted for sex. Blood pressure analyses were further adjusted for child height at the age of 8 years.

^2^Binary logistic regression odds ratio (95%CI) and p-values are presented, referenced to the mixed trajectory.

# Supplementary information

## Maternal characteristics

Information on mother’s age, ethnicity, educational attainment, household income, and pre-pregnancy weight was obtained from questionnaires administered at recruitment in the first trimester. Mother’s height was measured at 26–28 weeks’ gestation to calculate BMI. Family history of cardiovascular disease was obtained from questionnaires administered at recruitment and at postnatal year 4 and 8 visit.

## Paternal characteristics

Information on father’s age and educational attainment was obtained from questionnaires administered at postnatal year 2 or 3 visit. Father’s height and weight were measured during the visit to calculate BMI.

## Pregnancy characteristics

At 26–28 weeks’ gestation, information on diet quality (determined by Healthy Eating Index for pregnant women in Singapore) (1), moderate-to-vigorous intensity physical activity (≥ 150min/week) (2), television viewing time (< 2 h/day) (2), good sleep quality (determined by Pittsburgh Sleep Quality Index score >5) (3), and tobacco exposure (measured by plasma cotinine levels above the limit of detection ≥0.17 ng/mL) (4) was obtained.

Gestational diabetes was diagnosed using 2-h 75-g oral glucose tolerance tests based on the World Health Organization guideline (5). Depressive and anxiety symptoms were measured with the Edinburgh Postnatal Depression Scale (score ≥15) and State-Trait Anxiety Inventory (score ≥42) (6), respectively. Rate of gestational weight gain was determined by the Institute of Medicine guidelines according to pre-pregnancy BMI (7).

## Offspring characteristics

Information on infant sex, birth order, and birth weight was obtained from birth records. Gestational age was determined by first trimester ultrasound scan and infants born <37 weeks’ gestation were defined as preterm birth. We calculated sex- and cohort-specific birth weight percentiles (8) and defined small-for-gestational age (birth weight <10^th^ percentile), appropriate-for-gestational age (10^th^ to 90^th^ percentile), and large-for-gestational age (>90^th^ percentile).

## Postnatal characteristics

Duration of any breastfeeding was obtained from questionnaires administered at 3 weeks postnatally and at 3-month intervals from the ages 3 to 18 months. At the age of 9 months, age of introduction of first foods were collected retrospectively and early complementary feeding defined as complementary feeding initiated before 4 months of age (9).

References

1. Chia A-R, Tint M-T, Han CY, Chen L-W, Colega M, Aris IM, et al. Adherence to a healthy eating index for pregnant women is associated with lower neonatal adiposity in a multiethnic Asian cohort: the Growing Up in Singapore Towards healthy Outcomes (GUSTO) Study. The American Journal of Clinical Nutrition. 2018;107(1):71-9.

2. Padmapriya N, Shen L, Soh SE, Shen Z, Kwek K, Godfrey KM, et al. Physical Activity and Sedentary Behavior Patterns Before and During Pregnancy in a Multi-ethnic Sample of Asian Women in Singapore. Maternal and child health journal. 2015;19(11):2523-35.

3. Cai S, Tan S, Gluckman PD, Godfrey KM, Saw S-M, Teoh OH, et al. Sleep Quality and Nocturnal Sleep Duration in Pregnancy and Risk of Gestational Diabetes Mellitus. Sleep. 2016;40(2).

4. Ng S, Aris IM, Tint MT, Gluckman PD, Godfrey KM, Shek LP-C, et al. High Maternal Circulating Cotinine During Pregnancy is Associated With Persistently Shorter Stature From Birth to Five Years in an Asian Cohort. Nicotine & Tobacco Research. 2018:nty148-nty.

5. Alberti KGMM, Zimmet Pf. Definition, diagnosis and classification of diabetes mellitus and its complications. Part 1: diagnosis and classification of diabetes mellitus. Provisional report of a WHO consultation. Diabetic medicine. 1998;15(7):539-53.

6. Padmapriya N, Bernard JY, Liang S, Loy SL, Shen Z, Kwek K, et al. Association of physical activity and sedentary behavior with depression and anxiety symptoms during pregnancy in a multiethnic cohort of Asian women. Archives of Women's Mental Health. 2016;19(6):1119-28.

7. Rasmussen KM, Yaktine AL. Weight gain during pregnancy: reexamining the guidelines. 2009.

8. Mikolajczyk RT, Zhang J, Betran AP, Souza JP, Mori R, Gülmezoglu AM, et al. A global reference for fetal-weight and birthweight percentiles. The Lancet. 2011;377(9780):1855-61.

9. Ong YY, Pang WW, Michael N, Aris IM, Sadananthan SA, Tint M-T, et al. Timing of introduction of complementary foods, breastfeeding, and child cardiometabolic risk: a prospective multiethnic Asian cohort study. The American Journal of Clinical Nutrition. 2023;117(1):83-92.
